# A comparison of stress, symptoms, physical activity, and adiposity among women at midlife before and during the pandemic

**DOI:** 10.1186/s40695-022-00075-w

**Published:** 2022-04-05

**Authors:** Lynnette Leidy Sievert, Sofiya Shreyer, Ashley Boudreau, Sarah Witkowski, Daniel E. Brown

**Affiliations:** 1 Department of Anthropology, UMass Amherst, Machmer Hall, 240 Hicks Way, Amherst, MA 01003-9278 USA; 2 Exercise & Sport Studies, Smith College, Northampton, MA USA; 3 Anthropology, University of Hawaii at Hilo, Hilo, HI USA

**Keywords:** COVID-19, Menopause, Midlife, Stress, Depression, Physical activity

## Abstract

**Background:**

The COVID-19 pandemic presented challenges that disproportionately impacted women. Household roles typically performed by women (such as resource acquisition and caretaking) became more difficult due to financial strain, fear of infection, and limited childcare options among other concerns. This research draws from an on-going study of hot flashes and brown adipose tissue to examine the health-related effects of the COVID-19 pandemic among 162 women aged 45–55 living in western Massachusetts.

**Methods:**

We compared women who participated in the study pre- and early pandemic with women who participated mid-pandemic and later-pandemic (when vaccines became widely available). We collected self-reported symptom frequencies (e.g., aches/stiffness in joints, irritability), and assessments of stress, depression, and physical activity through questionnaires as well as measures of adiposity (BMI and percent body fat). Additionally, we asked open-ended questions about how the pandemic influenced women’s health and experience of menopause. Comparisons across pre-/early, mid-, and later pandemic categories were carried out using ANOVA and Chi-square analyses as appropriate. The Levene test for homogeneity of variances was examined prior to each ANOVA. Open-ended questions were analyzed for yes/no responses and general themes.

**Results:**

Contrary to our hypothesis that women would suffer negative health-related consequences during the COVID-19 pandemic, we found no significant differences in women’s health-related measures or physical activity across the pandemic. However, our analysis of open-ended responses revealed a bi-modal distribution of answers that sheds light on our unexpected findings. While some women reported higher levels of stress and anxiety and lower levels of physical activity, other women reported benefitting from the remote life that the pandemic imposed and described having more time to spend on physical activity or in quality time with their families.

**Conclusions:**

In this cross-sectional comparison of women during the pre-/early, mid-, and later-pandemic, we found no significant differences across means in multiple health-related variables. However, open-ended questions revealed that while some women suffered health-related effects during the pandemic, others experienced conditions that improved their health and well-being. The differential results of this study highlight a need for more nuanced and intersectional research on risk, vulnerabilities, and coping among mid-life women.

## Background

The United States has been in the grip of coronavirus disease (Covid-19) since its first confirmed case in January 2020. By the end of March 2020, just after the WHO declared Covid-19 to be a global pandemic, the United States led the world in confirmed cases with more than 1000 deaths. By December 2020, the death toll exceeded 300,000 in the United States, and the FDA approved the Pfizer and Moderna vaccines for emergency use [1]. By the end of February 2021, about 25 million people were fully vaccinated in the U.S. (7.5%) and about 500,000 individuals were fully vaccinated in the state of Massachusetts (7.2%), including nurses and other health care providers [2]. Vaccination rates rose quickly in the months of March and April 2021, reaching teachers and individuals with just one medical condition (including diabetes and overweight/obesity) [3].

The beginning of the pandemic was stressful for many reasons, including fears of contagion, constant media coverage, and inadequate supplies of basic necessities, face masks, and disinfectants. The pressure continued through 2020 into 2021 because of social distancing, self-quarantine, sickness, loss of loved ones, travel bans, and economic worry. Women are especially vulnerable during times of crises because they are frequently the primary resource managers for the household and caretakers for dependents, adjusting everyday life during a disaster to create a sense of security for their families [4]. Women often solve the challenges that arise in the household sphere, including limited resource availability, financial troubles, and medical assistance [5]. Disasters such as floods [6] and hurricanes [7] result in significantly higher levels of stress among women for multiple reasons. During Covid-19, women at midlife lost or left jobs because of furloughs or the demands of homeschooling. Others worked on the frontlines or shared a small workspace with partners and children. Some women protected the precarious health of their vulnerable parents with higher levels of caretaking. Still other women found themselves alone and isolated.

Pandemics and other large-scale disasters are almost always accompanied by increases in depression and behavioral disorders [8], and it appears that women have been more vulnerable to stress, depression, and anxiety during Covid-19 [9]. Extended self-quarantines due to Covid-19 have been associated with changes in nutritional habits [10, 11] and weight [12]. A cross-national study of 1047 individuals found that vigorous, moderate, and walking activity declined during home confinement, while hours individuals spent sitting for more than 8 h per day increased from 16 to 40% [13]. Other studies showed that individuals maintained better health-related quality of life during Covid-19 by living with others [14] and having a higher health literacy [15].

The research presented here integrated questions about Covid-19 into an ongoing study of hot flashes and brown adipose tissue among women aged 45 to 55 (*n* = 162) in order to take advantage of the secular timing of the pandemic and provide preliminary data on the impact of the pandemic on the health of midlife women. The timeframe of data collection provided the opportunity to compare stress, depression, symptoms at midlife, levels of physical activity, and adiposity across three periods of time – the pre-/early pandemic (October 2019 through March 2020, *n* = 36), the mid-pandemic (October 2020 through February 2021, *n* = 39) and the later pandemic (March 2021 to May 2021 and October 2021 to January 2022, *n* = 87). The divide between mid-pandemic and later pandemic is based on when vaccines became widely available to our population in Massachusetts. Our sample of women aged 45 to 55 included health care workers, teachers, and individuals with one medical condition who qualified for an early Covid-19 vaccine.

The purpose of this study was to examine health-related effects associated with Covid-19 among women in western Massachusetts, using quantitative and qualitative methods. We hypothesized that women sampled during the pandemic (mid- and later pandemic) would report higher levels of stress and depressed mood, more general symptoms, lower levels of physical activity, and a higher level of adiposity compared to women sampled before the pandemic and before home confinement began (pre-/early pandemic).

## Methods

These data were drawn from an on-going study of hot flashes and brown adipose tissue. Because brown adipose tissue is most active during cool weather [16], the study was designed to collect data each year from October through the beginning of May. For the research presented here, the first time period started during a pre-Covid year in October 2019 and extended to mid-March 2020, when the study was shut down because of the closing of the University due to the pandemic. Data could not be collected from mid-March to the beginning of May, and therefore a period of self-quarantine, confusion, and family upheaval was missed. The study began again in October 2020 with pandemic precautions in place, and the interview was shifted to Zoom in order to shorten the amount of time in the laboratory. This second time period (“mid-Covid-19”) extended until the end of February 2021. The third time period (“later Covid-19”) started in March 2021 and continued to May 2021. During this third period of time, many of the participants received at least one vaccination because they were health care workers, K-12 teachers, or had at least one health condition such as overweight, obesity or diabetes. The “later Covid-19” period of time also includes October 2021 to January 2022. Beginning in October 2021, interviews were again carried out in the laboratory, and the majority of participants had been vaccinated and received booster shots.

At first, participants were recruited to the study with brochures mailed to women aged 45 to 55 selected randomly from town clerk lists in western Massachusetts. We then recruited women aged 45 to 55 within a 20-mile radius of UMass Amherst with Facebook ads. We targeted late peri-menopausal women with irregular menstruation and early post-menopausal women within two years of their last menstrual period. The most important criterium was age, therefore some pre-menopausal women were included in the sample. Exclusion criteria included use of hormone therapy or other medications that dampen hot flashes.

A semi-structured questionnaire was administered in person (pre-pandemic and after October 2021) or by Zoom (October 2020 to May 2021) to collect demographic, reproductive, and lifestyle information (*n* = 162). The questionnaire included the question, “Thinking back over the past two weeks, have you ever been bothered by any of the following?” This was followed by 23 symptoms drawn from the list of Everyday Complaints [17] and the Greene Climacteric Scale [18]. Women responded with “not at all,” “a little,” “somewhat,” and “a lot.” This list of symptoms has been used in many studies [19, 20] and continues to be used for cross-cultural comparisons. For the research presented here, symptoms were analyzed as dichotomous yes/no categories.

Before and during the pandemic, participants came into the laboratory for body measurements, bioelectrical impedance measures, and for the estimation of brown adipose tissue via thermal imaging (*n* = 158). While in the laboratory, women independently filled out the Patient Health Questionnaire (PHQ-9) [21] as a measure of depression as well as the Perceived Stress Scale (PSS-10) [22]. Physical activity was assessed with the International Physical Activity Questionnaire Short Form (IPAQ-SF) [23]. Low, moderate, and high levels of physical activity were determined by cutoffs based on MET-minutes/week and number of days with combined vigorous-intensity, moderate-intensity, and walking activity (low: < 600MET-minutes/week; moderate: ≥ 600MET-minutes/week and < 3000MET-minutes/week; vigorous: ≥ 3000MET-minutes/week). From December 2020 to May 2021, two open-ended questions were added to the laboratory session: “Do you think the pandemic has influenced your health? How?” and “Do you think the pandemic has influenced your experience of menopausal symptoms, like hot flashes? How?”.

Height was measured with a Seca stadiometer to the nearest 0.1 cm. Weight was measured with an analog Health o meter scale to the nearest 0.1 kg. Body mass index (BMI) was computed as kg/m^2^. Percent body fat was calculated from bioelectrical impedance measures (RJL Prizum Systems, Clinton Township, MI).

Comparisons across pre-/early, mid-, and later pandemic categories for stress, depressed mood, and adiposity (BMI and percent body fat) were carried out using ANOVA. Prior to each ANOVA, homogeneity of variances was examined using the Levene test. Multiple linear regressions were carried out to assess the potential effect of covariates (parity, economic comfort, level of education, and employment) on PSS-10 and PHQ-9 scores. The five most common symptoms (aches/stiffness in joints, difficulty concentrating, irritability, hot flashes, and trouble sleeping) and categories of physical activity were compared across pre-/early, mid-, and later pandemic categories using contingency table (Chi-square) analyses. Open-ended questions were analyzed for yes/no responses and general themes.

Posthoc sensitivity analyses were carried out to determine estimates of Cohen’s effect sizes [24] based on measured sample sizes and degrees of freedom, along with α = 0.05 and power = 0.80. Our ANOVA effect size (f) was 0.25, and our Chi-squared effect size (w) was 0.24. Based on Cohen’s categories, this sensitivity analysis indicates that our sample size would allow us to detect medium-sized effects using ANOVA, and small to medium-sized effects using a Chi-squared test.

## Results

In general, the participants in this study were highly educated, married (72%), and identified as heterosexual (78%), with one or two children (62%). They were employed (87%) and economically “OK” or “comfortable” (83%) with “good” or “excellent” health (93%). They drank alcohol (77%) but did not smoke (4%). Across the three time periods, there were no significant differences in sample characteristics, as shown in Table 1.

**Table 1:** Sample characteristics by time period

	Total *n* = 162	Pre-/early Covid-19 *n* = 36	Mid- Covid-19 *n* = 39	Later Covid-19 *n* = 87	*p*-value^a^
Age at interview (years)	51.1 (2.9)	50.2 (2.4)	51.6 (3.1)	51.2 (2.9)	0.070
Level of education					
High school or less	4.9%	5.6%	10.3%	2.3%	0.145
Some or graduated college	46.3%	33.3%	48.7%	50.6%	
Some graduate school	48.8%	61.1%	41.0%	47.1%	
Marital status					
Single	13.0%	16.7%	10.3%	12.6%	0.905
Married/living together	72.2%	72.2%	76.9%	70.1%	
Separated/divorced	14.2%	11.1%	12.8%	16.1%	
Widowed	0.6%	0	0	1.1%	
Sexual orientation					
Lesbian or gay	11.1%	11.1%	5.1%	13.8%	0.407
Heterosexual	78.4%	72.2%	84.6%	78.2%	
Other	10.5%	16.7%	10.3%	8.0%	
Parity					
0	19.1%	13.9%	25.6%	18.4%	0.174
1	16.7%	13.9%	20.5%	16.1%	
2	45.1%	63.9%	33.3%	42.5%	
3 +	19.1%	8.3%	20.5%	23.0%	
Employed (%)	87.0%	91.4%	76.9%	89.7%	0.098
Economic comfort					
Struggling	6.9%	2.8%	2.7%	10.3%	0.299
OK	38.1%	33.3%	37.8%	40.2%	
Comfortable	44.4%	47.2%	54.1%	39.1%	
Well-off	10.6%	16.7%	5.4%	10.3%	
Self-reported health^b^					
OK	6.8%	5.6%	10.3%	5.7%	0.846
Good	51.2%	50.0%	46.2%	54.0%	
Excellent	42.0%	44.4%	43.6%	40.2%	
Drink alcohol (%)	76.5%	75.0%	74.4%	78.2%	0.870
Smoke (%)	3.7%	2.8%	7.7%	2.3%	0.315

^a^ *P*-value for differences across the three time periods

^b^ No participants chose the option of “Poor” to describe their health

**Fig. 1:**
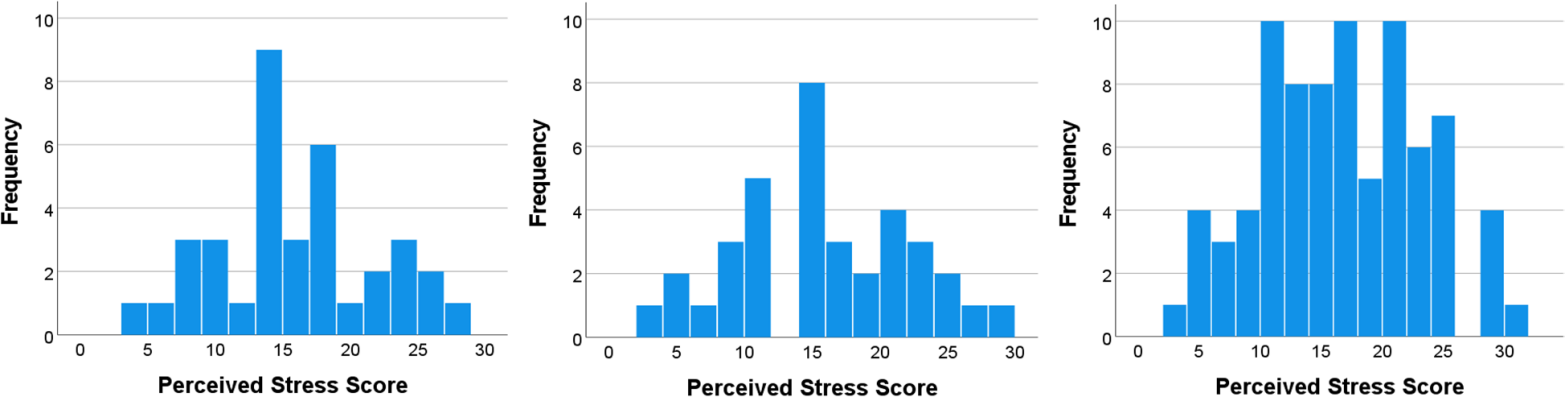
Histograms of frequencies of PSS-10 scores across pre/early, mid-, and later Covid-19 time periods, respectively, left to right

Across pre-/early, mid- and later pandemic categories, mean perceived stress scores (PSS-10) remained remarkably consistent (15.5 vs. 15.6 vs. 16.2, *p* = 0.820; Table 2 and Fig. 1), and population variances were similar (Levene’s test). Linear regression results also showed that the timeframe of Covid-19 was not associated with perceived stress scores. Only economic comfort was associated with perceived stress (Table 3).

**Table 2:** Perceived stress, depression, symptom frequencies, physical activity, BMI, and percent body fat by time period

	Total	Pre-/early Covid-19	Mid-Covid-19	Later Covid-19	*p*-value^a^
PSS-10^b^ score	15.9 (6.4)	15.5 (5.9)	15.6 (6.6)	16.2 (6.6)	0.820
PHQ-9^c^ score	4.9 (3.7)	5.0 (3.4)	5.2 (3.9)	4.8 (3.7)	0.829
*Symptom frequencies*					
Aches/stiffness in joints	81.5%	86.1%	82.1%	79.3%	0.673
Irritability	74.7%	72.2%	71.8%	77.0%	0.765
Difficulty concentrating	72.8%	72.2%	66.7%	75.9%	0.560
Hot flashes	71.6%	69.4%	71.8%	72.4%	0.946
Trouble sleeping	66.7%	63.9%	74.4%	64.4%	0.504
*Physical activity levels*					
Low physical activity	15.3%	19.4%	11.1%	15.3%	0.889
Moderate physical activity	44.6%	44.4%	44.4%	44.7%	
High physical activity	40.1%	36.1%	44.4%	40.0%	
*Measures of adiposity*					
BMI (kg/m^2^)	28.0 (6.1)	27.6 (6.7)	29.8 (5.8)	27.5 (6.0)	0.151
Percent body fat (%)	36.7 (7.8)	35.7 (7.4)	38.6 (7.8)	36.3 (8.0)	0.230

^a^ *P*-value for differences across the three time periods

^b^ Perceived Stress Scale-10 [22]

^c^ Patient Health Questionnaire-9 [21]

**Table 3:** Results of linear regressions for PSS-10^a^ and PHQ-9^b^ scores

PSS-10		
	Unstandardized B (SE)	*P*-value
Covid timeframes	0.207 (0.632)	0.744
Parity 0–3 +	0.561 (0.528)	0.289
**Economic comfort**	**-1.762 (0.701)**	**0.013**
Level of education	1.363 (0.910)	0.136
**PHQ-9**		
Covid timeframes	-0.300 (0.358)	0.403
Parity 0–3 +	0.259 (0.292)	0.376
**Economic comfort**	**-0.968 (0.380)**	**0.012**
Employed	1.415 (0.871)	0.106

^a^ Perceived Stress Scale-10 [22]

^b^ Patient Health Questionnaire-9 [21]

Covid timeframes coded as 1 = pre/early, 2 = mid, 3 = later. Parity coded as 0,1,2,3 + . Economic comfort coded as 1 = struggling, 2 = OK, 3 = comfortable, 4 = well-off. Level of education coded as 1 = high school, 2 = some college or college graduate; 3 = some graduate school or graduate degree. Employed coded as 1 = yes, 2 = no

**Fig. 2:**
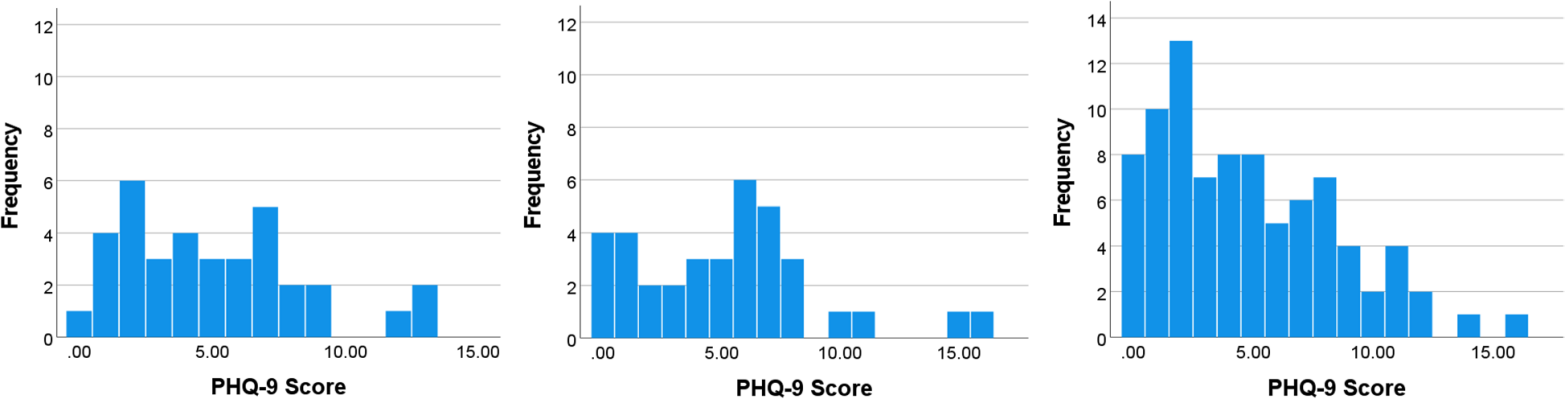
Histograms of frequencies of PHQ-9 scores across pre/early, mid-, and later Covid-19 time periods, respectively, left to right

Mean PHQ-9 scores also did not significantly differ across pre-/early, mid- and later pandemic categories (5.0 vs. 5.2 vs. 4.8, *p* = 0.829). Linear regression results confirmed that the timeframe of Covid-19 was not associated with PHQ-9 scores. Only economic comfort was associated with PHQ-9 scores (Table 3). Histograms (Fig. 2) show that more women scored below 5 in the later pandemic; according to Kroenke et al. [21], scores less than 5 almost always signify the absence of a depressive disorder. Using a cutoff of ≥ 10 [21, 25], women were not more likely to be moderately to severely depressed during the COVID pandemic.

Table 2 shows symptom frequencies across the three time periods. Aches/stiffness in joints (82%), irritability (75%), difficulty concentrating (73%), hot flashes (72%) and trouble sleeping (67%) were the most frequently reported symptoms. There were no significant differences in symptom frequencies across the pandemic categories using Chi-square analyses.

From the IPAQ-SF, time spent sitting on a weekday was similar across the pandemic timeframe (pre-/early pandemic 6.0 h (s.d. 2.9) vs. mid-pandemic 6.5 h (s.d. 3.0) vs. later pandemic 6.7 h (s.d. 3.5), *p* = 0.580). Neither were there significant differences in the hours/week spent in vigorous activity (2.4 h vs. 2.0 h vs. 2.8 h, *p* = 0.568), hours/week spent in moderate activity (6.4 h vs. 4.8 h vs. 3.2 h, *p* = 0.219), or hours/week spent walking (8.4 h vs. 7.9 h vs. 11.9 h, *p* = 0.681).

When activity levels were scored into low, moderate, and high categories, there were no significant differences by time period, using Chi-square analyses, as shown in Table 2. Neither were mean BMI (kg/m^2^) or percent body fat significantly different across the pandemic categories, using ANOVA, as shown in Table 2.

Open-ended answers reveal a bi-modal distribution of responses among women with regard to the pandemic and their health. During the mid-pandemic period (October 2020 to February 2021), the topic most often volunteered in response to the question, “Do you think the pandemic has influenced your health?” was activity level. Ten women described themselves as less active because they were working from home, not able to go to the gym, and “not training for anything.” In contrast, five women had more time to work out, took more hikes and bike rides, and experienced fewer disruptions while doing physical activity. One woman explained that she was “still doing the same things, work, grocery shopping, and stacking wood.”

The second most common topic volunteered during the mid-pandemic was stress and mental health. Eight women described stress related to jobs, inability to travel, and social isolation. Said one 53-year-old woman, “I’m feeling depressed and down. There’s not enough social interaction. It’s harder to get out the door, and I’ll work in my pajamas all day. I’m more fearful and anxious, irritated more easily.” On the other hand, two women felt less stressed during the pandemic. One 50-year-old participant explained her “mental health improved because of more time with family and business is prospering.” Another participant said that the pandemic hadn’t affected her stress level, either up or down.

The third most common topic during the mid-pandemic was eating and weight change. Five women said they gained unwanted weight, were overeating, and eating more junk food. In contrast, three women explained that they were eating “pretty healthy,” had lost weight, and had more time to focus on what they were eating. Table 4 shows additional topics volunteered by study participants, including improved self-care among four participants who felt they had more time to take better care of themselves and more time to sleep.

**Table 4:** Topics volunteered by study participants in response to the question “Do you think the pandemic has influenced your health?” (72 respondents)^a^

	Mid-pandemic responses (Compared to pre-pandemic baseline)	Later pandemic responses (Compared to pre-pandemic baseline)
Activity level	10 less activity, 5 more activity, 1 unchanged	13 less activity, 7 more activity
Stress and mental health	8 worsened, 2 improved 1 unchanged	9 worsened, 0 improved
Eating and weight change	5 worsened, 3 improved	5 worsened, 5 improved 1 unchanged
Self-care	1 worsened, 4 improved	1 worsened, 4 improved
Isolation	4 felt more isolated	7 felt more isolated
Alcohol intake	1 increased intake 1 decreased intake	(none)
Other infections	Haven’t caught typical colds	First winter without “an upper respiratory thing”

^a^ Qualitative question administered December 2020 to May 2021

The same themes emerged during the later pandemic (March 2021 to May 2021). Most responses focused on activity levels, with thirteen women describing how they stopped exercising and became more sedentary. One 50-year-old participant explained that she used to work at the Y. Without her job, she lost her membership. She is now sitting more and exercising less. On the other hand, seven women described having more time to exercise. One participant explained how she was “not driving so much,” so she could work out more.

The second category of responses was about eating and weight change. This category was evenly split between women gaining and losing weight. A 50-year-old participant explained that her father quarantined with her; he likes wine and appetizers with dinner; and she gained 20 pounds to reach her highest weight. In contrast, a 54-year-old participant said she lost weight because she’s eating less drive-through food. A 55-year-old participant said, “In the beginning, we sat and gained weight, made dessert almost every night. Now, I think about my eating, I’ve lost weight.”

The third most common set of responses during the later pandemic referred to stress and mental health. No one volunteered improved mental health. Women detailed the stress of taking care of an elderly mother, changing work duties, and the disruption of everyday routines. One participant talked about the “depressive energy of Covid.” A 54-year-old participant said that she had “a lot more stress. Lost a job. And it’s scary to go grocery shopping.”

Isolation was an often-reported concern, with women describing how they lost touch with people, missed their families, were unable to engage in community, felt more cautious about people, and found it hard to get out and go places. In contrast, one 51-year-old participant described, “we’re doing what we’re supposed to do, not so much work, work, work. My house feels more like home because I’m there all the time.”

Table 4 shows additional themes, including self-care. For example, one 53-year-old participant described herself as “very motivated.” Her self-care during the pandemic included diet and gym, “then a Fitbit, then a treadmill, now yoga.” Only four (6%) of the 72 women questioned thought that the pandemic had not influenced their health.

The majority of women (64%) said they did not think the pandemic influenced their experience of menopausal symptoms. Those who felt their hot flashes were less frequent (*n* = 3) attributed the change to drinking less and lower levels of stress. Those who felt their hot flashes were more frequent or more severe attributed the change to increased awareness or having more time to notice hot flashes (*n* = 7), experiencing a higher level of stress (*n* = 9), lack of exercise (*n* = 2), and drinking more alcohol (*n* = 1). One 53-year-old participant described more hot flashes due to wearing an N95 mask and goggles all the time at work. Another 53-year-old participant with severe hot flashes said she was more aware of hot flashes because she was not in the car as often. In the car, she was “able to turn on AC. It’s harder to open a window at home.”

## Discussion

The purpose of this study was to examine health-related outcomes potentially associated with the Covid-19 pandemic among midlife women in western Massachusetts. We hypothesized that women sampled during the mid- and later pandemic would report higher levels of stress and depressed mood, more general symptoms, lower levels of physical activity, and a higher level of adiposity compared to women sampled during the pre- and early pandemic. Our hypotheses were not supported. Comparisons across pre-/early, mid-, and later pandemic categories did not reveal higher levels of stress, depression, symptom frequencies, sedentary behavior, or adiposity in association with the life changes brought about by Covid-19. The comparisons involved different samples of women, but there were no significant differences across the three samples.

This study presented an opportunity to make comparisons across three time periods, with questions asked in the same way both before and during the pandemic. It is noteworthy that the mean PSS and PHQ-9 scores stayed so consistent. We gave particular attention to Levene’s test for homogeneity in order to test for differences in population variances, but they did not differ. We examined histograms for bimodality in the distribution of scores.

Aches/stiffness in joints, irritability, difficulty concentrating, hot flashes, and trouble sleeping were the most frequently reported symptoms; however, there were no significant differences in symptom frequencies across the pandemic categories.

Consistent with the findings of Ammar et al. [13], hours spent sitting were higher across the pre-/early, mid-, and later pandemic categories (n.s.). Activity levels scored as low, moderate, and high did not differ by time period. Mean BMI (kg/m^2^) and mean percent body fat also were consistent across the pandemic categories.

The consistency of results from comparisons of means and frequencies is all the more remarkable when we read the open-ended responses to the question “Do you think the pandemic has influenced your health?” Here the answers were often bimodal. During the Covid-19 pandemic in Chile, Reyes-Olavarría et al. [11] described how weight increased for some women (38%) but declined for others (14%); how physical activity increased for some women (20%) but declined for others (59%). In western Massachusetts, women described themselves as less active or more active, losing weight or gaining weight, practicing self-care or increasingly poor health habits. Participants gave explanations for their increased feelings of stress and the “depressive energy of Covid,” but also shared positive points of view.

An interesting topic that surfaced from qualitative responses was the centrality of driving. During the pandemic, women drove less often. That made more time for exercise and reduced the consumption of drive-through meals. However, less time in the car also took away the air conditioning that helps with hot flashes.

Limitations include the cross-sectional study design. We compared three different groups of women at three different points in time. Also, our sample lacks diversity. Because of the location of our work in western Massachusetts, we have a largely white population of middle-class women, the majority of whom were vaccinated as soon as the vaccines were available. We did not systematically ask women if they were vaccinated, but when we volunteered that we were vaccinated, almost all women volunteered their own vaccination status (one, two, or three shots) in return. Finally, there was a selection for women willing to visit a college campus to participate in a research study during a pandemic. These women may not be representative of the general Massachusetts population and may differ in study-relevant ways from those recruited before the pandemic.

The strength of the study was that we started to ask the questions about stress, depression, symptoms at midlife, and physical activity, and started to take body measurements, before Covid-19 was on the horizon. We continued to ask the same questions and take the same body measurements throughout the pandemic until January 2022. We were able to add two open-ended questions to the study to assess how women felt the pandemic influenced their health. This mixed methods approach suggested that the comparisons of means and frequencies did not capture the results of what might be bimodal effects of the pandemic on women’s experience. Future research should explore divergent effects and means of coping among women at midlife.

## Conclusions

This cross-sectional comparison of women at midlife showed, contrary to our hypotheses, that there were no significant differences in multiple health-related variables at three different time periods during the COVID-19 pandemic. Qualitative results suggest that while some women experienced ill health effects, others experienced conditions that allowed better health during this unprecedented time. The study highlights issues that are important for understanding the impact of the pandemic and the variation in women’s experience.

## Data Availability

The datasets used and analyzed during the current study are available from the corresponding author on reasonable request. This is an on-going study. At completion, data will be available through the Scholarworks site of UMass Amherst.
